# Association between antimicrobial usage, biosecurity measures as well as farm performance in German farrow-to-finish farms

**DOI:** 10.1186/s40813-018-0106-5

**Published:** 2018-12-14

**Authors:** S. Raasch, M. Postma, J. Dewulf, K. D. C. Stärk, E. grosse Beilage

**Affiliations:** 1 Field Station for Epidemiology, University of Veterinary Medicine Hannover, Buescheler Straße 9, 49456 Bakum, Germany; 2 Veterinary Epidemiology Unit, Department of Reproduction, Obstetrics and Herd Health, Faculty of Veterinary Medicine, Ghent University, Salisburylaan 133, 9820 Merelbeke, Belgium; 3 SAFOSO AG, Waldeggstrasse 1, CH 3097 Bern Liebefeld, Switzerland

**Keywords:** Pig production, Antimicrobial usage, Treatment incidence, Risk factors, Herd size, Biosecurity

## Abstract

**Background:**

Antimicrobial usage in food producing animals is of major concern. A clear link between the extent of use and the development of antimicrobial resistance has already been demonstrated. To evaluate strategies that may reduce the antimicrobial usage while assuring pig health and welfare, it requires profound knowledge of factors that are associated with antimicrobial usage. Data on biosecurity and herd management practices are important parameters to identify risk factors which are related to a higher antimicrobial usage. To investigate between-farm variations of high and low usage the treatment incidence (TI) per age group in 60 German farrow-to-finish herds was qualitatively and quantitatively analysed and linked to biosecurity measures, and herd management characteristics.

**Results:**

Weaned pigs received most of the treatments (median TI = 487.6), followed by suckling pigs (median TI = 138.9). Suckling pigs were treated with critically important antimicrobials (3rd and 4th generation cephalosporines) to a remarkable extent. The number of sows present at site (*p* < 0.01) and a low score for external biosecurity (*p* = 0.06) were associated with a higher antimicrobial usage in pigs from birth till slaughter. Herds with a higher treatment incidence in growing pigs (TI 200 days): i) were located in a region with a high pig density (*p* < 0.01), ii) had a less strict access check for visitors and personnel (*p* < 0.01) and iii) scored lower in the subcategory ‘cleaning and disinfection’ (internal biosecurity) (*p* < 0.01). Herds with a higher treatment incidence in breeding pigs weaned more piglets per sow and year and scored better in the internal biosecurity level (*p* = 0.02).

**Conclusions:**

With the main focus on the treatment incidence in pigs from birth till slaughter and in breeding pigs risk factors for a high usage in these age groups were identified. The level of biosecurity of a herd was associated with the amount of antimicrobials used. Therefore, the findings in this study indicate possible points of action in the reduction and prudent use of antimicrobials in Germany. The active improvement of biosecurity measures could be a promising alternative to reduce antimicrobial usage on herd level.

**Electronic supplementary material:**

The online version of this article (10.1186/s40813-018-0106-5) contains supplementary material, which is available to authorized users.

## Background

The modern pig production has grown to an efficient, economically driven industry in the EU (European Union). Farmers and veterinarians face various challenges to decrease the risk for disease in this intensive livestock production system. To prevent mortality and morbidity antimicrobials are an essential tool to treat bacterial infections. But this therapy option to cure bacterial infections in veterinary and human medicine is impeded due to the risk of selection for and spread of antimicrobial resistance [1]. A clear link between the extent of use and the development of antimicrobial resistance has already been demonstrated [2–5]. In countries with a high antimicrobial usage (AMU), high levels of antimicrobial resistance occurred [4]. International organisations such as the EMA (European Medicines Agency), WHO (World Health Organisation) and the OIE (World Organisation for Animal Health) are strongly recommending the implementation of monitoring activities. Hence, several EU countries are already conducting monitoring programmes for AMU [6–9]. Furthermore, the European Food Safety Authority (EFSA) and the EMA released a Joint Scientific Opinion upon measures to reduce AMU in animal husbandry and their impact on antimicrobial resistance [10]. This plan emphasises the need of a holistic, multidisciplinary approach to reduce AMU in all sectors related to public health. Germany, with a total population of 28 million pigs is one of the biggest pig producing countries in the EU [11]. The absolute amount of antimicrobials used is correspondingly high. One of the key elements to progress towards corrective actions is to establish a basis for a comprehensive understanding of extended AMU [12]. Reports on AMU over several EU countries already exist [13]. These reports however use national sales data of antimicrobials in kilogrammes or tons of active substance and relate these to the biomass of the animal population in the different reporting EU countries. Thus, a reallocation to a specific species is not possible. Besides the aforementioned limitation, the potency of an antimicrobial product is not considered in this quantification method [14].

Standards have been developed to measure AMU in a consistent approach to explain differences in the extent of and reasons for usage between EU member states [13]. The ESVAC (European Surveillance Veterinary Antimicrobial Consumption) report already provides data on sales of antimicrobials for food producing animals but does not give insight on the distribution by species and age. Just recently a study using a representative data set on the AMU provided details on the quantitative and qualitative use in pig herds in Germany [15]. The results showed that there are significant differences in antimicrobial classes prescribed and in the age pigs receive an antimicrobial treatment. The treatment incidence (TI) based upon the “defined daily dose” (DDD) can be calculated, if detailed information on herd level on AMU (e.g. total amount of antimicrobials administered), as well as information on the period at risk of receiving a treatment and the number of animals being treated at their respective weights are provided [16–18]. Harmonized data collection is a crucial starting point to explore association between management factors, production parameters, preventive measures and AMU on herd level. In order to progress towards corrective actions, it is necessary to further investigate reasons for different usage patterns on different farms and in different age groups and further explore risk factors that are associated with a higher usage on herd level.

According to several studies, the AMU varies between herds [1, 5, 16–19]. These variations may be related to differences in the herd health status, which is partly influenced by herd characteristics like different perspectives on biosecurity measures and disease preventions [12]. The improvement of biosecurity measures is an important approach to prevent the entry and spread of pathogens in a herd and thus may reduce the necessity of AMU [18, 19]. In order to differentiate the biosecurity level of a certain herd, a comprehensive and quantitative description of the level of biosecurity is necessary. The risk-based biosecurity scoring system (Biocheck.Ugent™) converts questions regarding the biosecurity practices of a certain herd into a score for its external (measures to prevent the introduction of pathogens into a herd from outside), internal (measures to reduce the spread of pathogens within a herd) and overall biosecurity status [20]. The results can be used to inform farmers and veterinarians on possible areas for improvement of the biosecurity level and provide a benchmarking by comparing the individual herd with other herds that had already been evaluated by Biocheck. Accordingly, this benchmarking method may serve as an effective tool to stimulate the reduction of AMU.

The aim of this cross-sectional study was to identify risk factors which contribute to a high AMU in 60 German farrow-to-finish herds. As a first step, the AMU was described in a qualitative and quantitative manner using the TI and a consensus “defined daily dose animal” (DDDA) [21]. Subsequently, the biosecurity measures and herd characteristics were analysed for their associations with the AMU.

## Materials and methods

### Herd selection

The cross-sectional study was performed in three regions with intensive pig production; Niedersachsen, Nordrhein-Westfalen and Mecklenburg-Vorpommern. These regions represent approximately 64% of the total pig production in Germany [22]. Only herds with at least 100 breeding sows and 500 fattening pigs present were included in the study. To allow comparison between the herds only full-line (sows and fattening pigs present in one location) or semi full-line herds (sow herds with 1:1 relation with the fattening pig herd at a different location) were asked to participate. Volunteering farmers were reached by different methods. The project was presented to consultancy circles for pig owners and to farmers’ associations. Moreover, veterinarians specialised in pigs were asked to encourage clients fulfilling the inclusion criteria. Finally, 60 herds were enrolled in the study.

### Herd visits and data collection

The herds were visited once on a convenient day between December 2012 and January 2014. All herds were examined by one and the same investigator following a standardised protocol. All participants were asked to sign a contract, where anonymity of all collected data was guaranteed. During the herd visit a questionnaire on paper was completed by interviewing the farmer. Farms were consecutively numbered (D1-D60) in order to anonymise the questionnaire. The questionnaire was basically divided into two parts. The first part included 21 open and closed questions concerning general herd characteristics (e.g. number of sows, number of full-time employees), antimicrobial treatments, disease incidences, management practices and vaccination protocols. Reproduction data were collected from the herd management system or provided by the farmers’ associations. For the information on the disease incidence the farmers were asked for the frequency of treatments (e.g. antimicrobials, electrolytes, probiotics, etc.) against certain disease symptoms per age category. For the suckling, weaned and fattening pigs, five disease symptom categories were defined comprising disorders of the locomotive system (e.g. lameness), gastro-intestinal tract, respiratory tract, central nervous system and skin. For the sows two more categories summarising diseases of the reproductive tract and the udder were added. The answers were transferred to a five-category-scale, where one was equal to never, two was rarely, three occasionally, four regularly and five commonly/always. To describe the workload per employee the number of breeding pigs per employee (full-time equivalent) was calculated. The number of pathogens vaccinated against was created by summing up all vaccinations used in a herd (for sows, boars, gilts, suckling, weaned and fattening pigs). For combined vaccines the single pathogens were accounted separately. The second part of the questionnaire included 109 questions concerning the biosecurity status of the herd (available online http://www.biocheck.ugent.be). All information on the herd management and biosecurity level corresponded to the twelfth months preceding the herd visit. The herd visit was completed by an inspection of the herd in order to validate the answers given during the interview.

For quantitative and qualitative data on the antimicrobial consumption the application and dispensing records on the AMU of the preceding twelfth months from every age and production group were collected. These records are statutory documents for veterinarians and farmers and were either provided by the farmer (copy or photograph) or the herd veterinarian (copy or E-mail) after receiving the permission to provide these records [23]. Besides information on the date of delivery, age group, identity of the animals, product name, purchased volume, dosage per animal, treatment duration, these forms specified the indication for the treatment. These variables are demanded by national law [23]. Commercial in-feed medication was not applied in the participating farms. Antimicrobial consumption data was anonymised using consecutive numbers (D1-D60). The assigned number was identical to the number in the questionnaire. The collected data was later entered in Excel 2010® (Microsoft Corporation).

### Quantification of antimicrobial usage

The application and dispensing records provided by the herd veterinarians and farmers were used as an input for the web based tool “ABcheck.UGent”, developed by Ghent University, Belgium. First, the system converted the amount of antimicrobials (in ml, l, g or kg) to active substance, expressed in milligram. Subsequently, it quantified the AMU for a specific herd by using the formula first described by Timmerman et al. [16] which included the period at risk, a standardised weight of the animals, the number of animals at risk and a consensus DDDA [21]. The obtained value was the treatment incidence (TI) which is a technical unit of measurement quantifying the number of animals out of a theoretical group of 1000 animals receiving a daily treatment with antimicrobials. This is equivalent to the number of days an animal would have been treated with antimicrobials, if it lived for a theoretical period of 1000 days. The TI was calculated using the following formula [16]:

$$ \mathrm{TI}=\frac{\mathrm{total}\ \mathrm{amount}\ \mathrm{of}\ \mathrm{antimicrobials}\ \mathrm{administered}\ \left(\mathrm{mg}\right)}{\mathrm{DDDA}\ \left(\frac{\mathrm{mg}}{\mathrm{kg}}\right)\ast \mathrm{number}\ \mathrm{of}\ \mathrm{days}\ \mathrm{at}\ \mathrm{risk}\ast \mathrm{number}\ \mathrm{of}\ \mathrm{animals}\ \mathrm{at}\ \mathrm{risk}\ast \mathrm{uniform}\ \mathrm{weights}\ \mathrm{for}\ \mathrm{specific}\ \mathrm{age}\ \mathrm{category}\ \left(\mathrm{kg}\right)}\ast 1000\ \mathrm{pigs}\ \mathrm{at}\ \mathrm{risk} $$

The numerator represents the total amount of antimicrobials administered in milligrams (mg) of active substance, which was applied to a certain group of animals in a defined period (denominator). The DDDA’s used in this study originated from a consensus DDDA list, which was previously developed by Postma et al. [21]. All antimicrobial products licenced until December 2013 were classified according to the WHO Anatomical Therapeutic Chemical (ATCvet) system. The days at risk for each age category were defined as the time period a pig could receive an antimicrobial treatment. For breeding animals, it was set to 365 days, for suckling, weaned and fattening pigs the rearing periods of the individual herds were used. Combined TI’s were calculated for breeding pigs (TI breeding pigs: sows, gilts and boars) and for pigs from birth till slaughter (TI 200 days: suckling, weaned and fattening pigs). The period at risk for pigs from birth till slaughter was standardised to a 200 days period to correct for differences in ages at slaughter between herds. For the calculation of the TI 200 days, the sum of the TI suckling pigs, weaned pigs and fattening pigs was divided with the number of days for the actual rearing period and finally multiplied with 200. Thus, the comparison of AMU between the herds was facilitated. To enhance comparability uniform weights for the age categories (in kilogrammes) were applied; 2 kg for suckling pigs, 7 kg for weaned pigs, 35 kg for fattening pigs, 60 kg for gilts and 220 kg for sows. Because of the different activity spectrum, the beta-lactams were evaluated separately in two groups: aminopenicillines and benzylpenicillines. Furthermore, graphs on the proportion of the used antimicrobial classes per age group and administration route were prepared to visualize the results.

### Quantification of the biosecurity status

For assessing the biosecurity status of a herd in a standardised way, the already validated risk-based biosecurity quantification tool “Biocheck.UGent™” was used [20]. This tool calculates a score for external, internal and overall biosecurity and thus allows a comparison of the biosecurity status of herds. Both parts are divided into six subcategories, each consisting of two to 13 mainly dichotomous and trichotomous questions. For external biosecurity these are: 1. purchasing policy (e.g. pigs from same supplier, health status documented, quarantine period), 2. removing animals, manure, carcasses, 3. supply fodder, water, equipment, 4. access check (e.g. availability of hygiene lock, strict separation of dirty and clean area in hygiene lock), 5. vermin, bird control, 6. location, environment (e.g. herd located in an area with high density of pigs [average pig density at municipality level > 300 pigs/km^2^], spotting of wild boars). For internal biosecurity, these subcategories are: 1. management diseases (e.g. availability of hospital pens), 2. farrowing, suckling period (e.g. frequency of cross-fostering of suckling pigs, frequency of manipulating [vaccination, castration] suckling pigs), 3. nursery period (e.g. all-in/all-out-management, mixing of different age groups), 4. fattening period, 5. compartmentalizing, working lines, equipment and 6. cleaning, disinfection. In the system every answer has a specific weight, depending on its importance in disease prevention. In case of absence of a biosecurity measure the score will be zero whereas in the presence of specific measures a score from 0.5 to ten is given (see detailed information on the scoring system in the supplementary files by Laanen et al. [20]). The weights of the questions were subsequently defined in a score for each subcategory, for the internal and external biosecurity and a total score, consisting of both internal and external scores, each ranging from zero (total absence of any biosecurity measure) to 100 (perfect biosecurity).

### Statistical analysis

Data on herd characteristics (e.g. management, technical parameters), biosecurity status, disease incidence and AMU were first managed using Excel 2010® (Microsoft Corporation). The results were analysed using descriptive statistics in SPSS Statistics (IBM SPSS 22.0, USA). For continuous variables the mean, median, minimum and maximum value and standard deviation were assessed. If continuous variables were not normally distributed a logarithmic or square root transformation was performed. In order to identify differences between low and high AMU, farms below the median TI 200 days (group 1) and above the median 200 days (group 2) were compared in terms of biosecurity scores using an independent sample t-Test with a significance level of 5% (*p* < 0.05). The threshold value used to define low versus high TI 200 days was the median TI 200 days of all participating farms (*n* = 60).To identify possible associations between AMU, herd characteristics, technical parameters and biosecurity status, the focus was laid on two specific predictor variables (outcome variables), namely the TI breeding pigs and the TI 200 days. The variables (risk factors), which might influence AMU or were being influenced by AMU, were then tested using univariable and subsequently multivariable linear regression models. The variables with *p* < 0.2 in the univariable model were retained for the multivariable model. To avoid multicollinearity, correlation between retained variables was assessed by means of the Pearson’s two-way-correlation coefficient and if the coefficient was > 0.6, only the best fitting variable was included in the multivariable model. The model was built by means of a manual stepwise backward selection procedure. During the modelling process confounding effects were evaluated by reviewing changes in parameter estimates. Additionally, residuals of the model were visually tested for homoscedasticity. All interaction between significant (*p* < 0.05) variables in the final model were tested.

## Results

### Herd characteristics

The median number of sows was 300 (range 100–1510) (Table 1). The median weaning age was 25.2 days (range 19.3–32.6 days) and the median number of weaned pigs per sow per year was 26.9 (range 21.1–32.2). A median of 107 breeding pigs (sows) per employee (range 50–450) was calculated. Most of the farms had a batch farrowing system of three-weeks (28/60), followed by one-week (16/60), two-weeks (10/60), four-weeks (5/60) and five-weeks (1/60).

**Table 1:** Descriptive information on the herd characteristics and technical parameters of 60 farrow-to-finish herds in Germany

Parameter	N	Mean	Median	SD	Min	Max
Number of sows/ herd	60	396	300	299	100	1510
Number of litters/ sow/ year	60	2.36	2.37	0.88	2.12	2.53
Mortality till weaning (%)	60	15.1	15.3	5.5	3.3	30.4
Weaning age (days)	60	24.4	25.2	3.3	19.3	32.6
Number of weaned pigs/ sow/ year	60	27.3	26.9	2.6	21.1	32.2
Number of sows per employee	58^a^	130	107	64	50	450
Number of slaughtered pigs/ year	60	5306	3740	5657	1000	40,000
Average daily weight gain (g/day)	38^a^	811.2	800	43.8	720	900
Average feed conversion ratio (g/g)/ year	27^a^	2.75	2.72	0.13	2.4	2.95
Years experience farmer	60	24.9	25	10.2	5	45
Number of pathogens vaccinated against	60	6.9	7	1.5	4	10

^a^Data was not available in all participating herds

### Antimicrobial usage and disease incidence

**Fig. 1:**
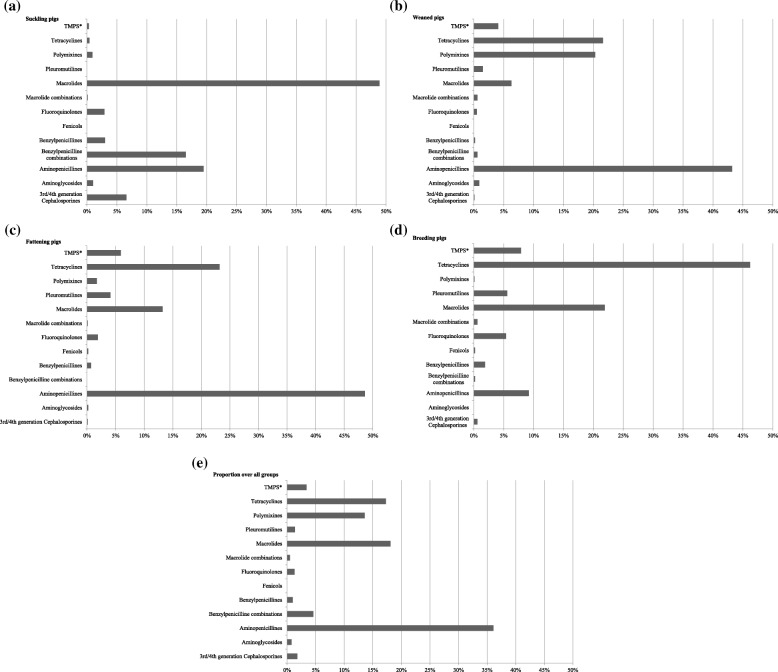
Proportion of the antimicrobial classes administered to suckling, weaned, fattening and breeding pigs. *TMPS: Sulfonamides and trimethoprim. **a** Suckling pigs **b** Weaned pigs **c** Fattening pigs **d** Breeding pigs **e** Proportion over all groups

**Fig. 2:**
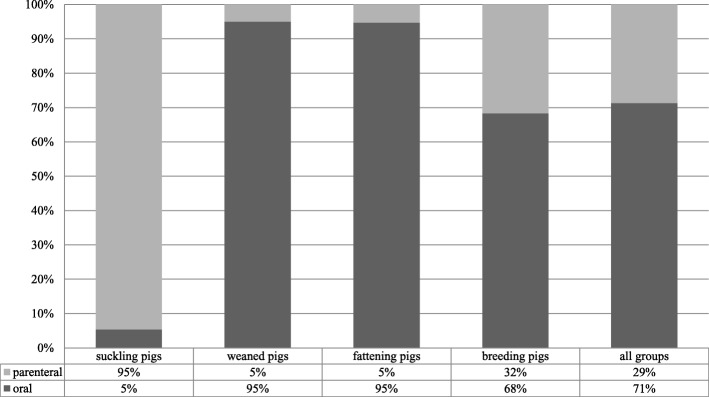
Proportion of the administration route (oral and parenteral) of the antimicrobial treatments (treatment incidence)

Most of the antimicrobial treatments were administered to suckling and weaned pigs with a median TI of 138.9 (range 8.1–1496.4) and 487.6 (range 8.5–1965.8), respectively (Table 2). Median TIs for fattening pigs and breeding pigs (sows, gilts and boars) were 51.7 and 42.0 per 1000 days at risk (ranges 0.0–399.2 and 0.2–204.5), respectively. The median treatment incidence for pigs from birth till slaughter, expressed as “TI 200 days”, was 242.8 (range 3.8–673.9). This means that pigs from birth till slaughter were treated with a daily dose of antimicrobials for 48.5 days out of the 200 days of their expected lifespan (Table 2). By the time of the AMU data collection a total of 281 generic products containing antimicrobial substances were licenced for pigs in Germany [21]. Suckling pigs were mainly treated with macrolides (49%) (Fig. 1a) and weaned pig received aminopenicillines (43%) the most (Fig. 1b). Moreover, in suckling pigs the 3rd and 4th generation cephalosporines (7%) and in weaned pigs the polymyxines (20%) were used in a noteworthy amount. Among the fattening pigs, aminopenicillines (49%) were mostly administered (Fig. 1c). The gilts and sows were mostly treated with tetracyclines (46%) (Fig. 1d). Over all age categories, aminopenicillines (36%), macrolides (18%) and tetracyclines (17%) were administered the most (Fig. 1e). From all antimicrobial substances used, 71% were administered orally, while 29% were given by injection (Fig. 2). The count and proportion of the five-category-scale for the frequency of treatment against defined disease symptoms per age category are displayed in an additional file (see Additional file 1). Treatments against lameness (occasionally: 42%; regularly: 14%) and gastro-intestinal (occasionally: 28%; regularly: 20%) diseases were common in suckling pigs. Weaned pigs were commonly treated against lameness (occasionally: 50%; regularly: 7%) and gastro-intestinal (occasionally: 28%; regularly: 27%) diseases, but also against respiratory diseases (occasionally: 48%; regularly: 10%). Fattening pigs were mostly treated against lameness (occasionally: 27%; regularly: 3%), respiratory (occasionally: 28%; regularly: 5%) and skin (occasionally: 28%; regularly: 5%) related symptoms. Breeding pigs had more reported treatments due to lameness (occasionally: 45%; regularly: 7%), reproductive (occasionally: 30%; regularly: 5%) and mastitis (occasionally: 38%; regularly: 12%) symptoms (see Additional file 1).

**Table 2:** Descriptive information on the treatment incidence per age category

Parameter	N	Mean	Median	SD	Min	Max
TI suckling pigs	60	245.0	138.9	257.4	8.1	1496.4
TI weaned pigs	60	633.4	487.6	491.5	8.5	1965.8
TI fattening pigs	58^a^	51.7	19.4	77.1	0.0	399.2
TI 200 days^b^	60	242.8	189.1	170.5	3.8	673.9
TI breeding pigs (gilts, sows, boars)	60	42.0	21.1	49.1	0.2	204.5

^a^Two farms with missing values for the TI fattening pigs

^b^The treatment incidence is defined as the number of pigs per 1000 pigs that receive a daily dose of antimicrobials. A TI 200 days is applied for pigs from birth till slaughter for comparison between herds, assuming an expected lifespan of 200 days

### Biosecurity scores

The biosecurity score in the studied herds showed variation in the different sub-categories and in the total scores (Table 3). Compared to the median of the total internal biosecurity (median = 55, range 37–82), the total score for external biosecurity was higher (median = 70, range 50–93). Among the external biosecurity sub-categories, the highest median score was achieved for “purchasing policy” with a score of 88 (range 30–100). The lowest score was reached for “location and environment” with a score of 30 (range 0–100). For internal biosecurity the lowest scores were obtained for “compartmentalizing, working lines, equipment” with 39 (range 11–100) and “cleaning and disinfection” with 45 (range 0–88). The highest score was seen for “fattening period” with 79 (range 21–91) and “nursery period” with 71 (range 31–100).

**Table 3:** Descriptive information on the biosecurity scores obtained in Biocheck.UGent™ on external and internal biosecurity and their specific subcategories

Parameter	N	Mean	Median	SD	Min	Max
External	60	70	70	9	50	93
Purchasing policy	60	84	88	14	30	100
Removing animals, manure, carcasses	60	79	79	10	50	100
Supply fodder, water, equipment	60	47	46	14	27	90
Access check	60	71	71	16	35	100
Vermin, bird control	60	71	70	19	30	100
Location, environment	60	38	30	31	0	100
Internal	60	55	55	9	37	82
Management diseases	60	64	60	25	40	100
Farrowing, suckling period	60	52	50	21	0	93
Nursery period	60	73	71	17	36	100
Fattening period	60	79	79	17	21	93
Compartmentalizing, working lines, equipment	60	41	39	15	11	100
Cleaning, disinfection	60	43	45	19	0	88

### Risk factors associated to a higher antimicrobial usage

Due to a right skewedness a LOG (logarithmic) transformation of the TI breeding pigs and the number of sows was needed to fit into the regression models. For the outcome variable TI 200 days a square root transformation was performed.

Farms belonging to the group 1 (below the median TI 200 days) scored significantly better for some biosecurity practices: in the external biosecurity they had better biosecurity practices on personnel and visitors (access check: *p* < 0.01) and were located in a more favorable region (location and environment: *p* < 0.01) with a for example lower pig density (i.e. average pig density at municipality level > 300 pigs/km^2^). In the internal biosecurity group 1 scored significantly better in cleaning and disinfection (*p* < 0.01) (Table 4). Moreover, the TI 200 days was significantly higher in larger herds (*p* = 0.02) (herd size expressed as the number of breeding pigs per herd) (Table 5). A lower TI 200 days was associated with a higher score for external biosecurity (*p* = 0.06). Regarding the treatment incidence of breeding pigs, two factors showing a significant effect (*p* < 0.05) on the TI breeding pigs were retained in the multivariable model. The number of weaned pigs per sow per year (*p* = 0.03) and the internal biosecurity (*p* = 0.02) were positively associated with a higher treatment incidence in the breeding sows (Table 5). Herds with a higher AMU in the breeding pigs weaned more pigs per sow per year and scored better in the internal biosecurity (adjusted r^2^ = 0.17).

**Table 4:** Comparison of the treatment incidence (mean and standard deviation) and biosecurity scores (two-sample t-Test for equal means) of farms below (*n* = 30) and above (*n* = 30) the median TI 200 days (186.1)

	Group 1: Farms < median TI 200 days (*n*^a^=30)	Group 2: Farms > median TI 200 days (*n*^a^=30)	*P*-value^3^
Treatment incidence	Mean (SD^b^)		
TI suckling pigs	162.0 (185.1)	328.2 (297.5)	
TI weaned pigs	284.4 (168.5)	982.4 (468.5)	
TI fattening pigs	21.9 (32.0)	84.0 (95.1)	
TI 200 days	113.0 (53.0)	372.6 (146.4)	
TI breeding pigs	45.0 (53.0)	39.2 (46.0)	
Biosecurity scores	Mean (SD^b^)		
External biosecurity			
Purchasing policy	87.9 (10.0)	88.1 (8.8)	0.94
Removing animals, manure, carcasses	80.7 (9.8)	77.5 (11.1)	0.24
Supply fodder, water, equipment	48.7 (15.2)	43.5 (12.4)	0.15
Access check	76.7 (15.3)	65.9 (15.0)	**< 0.01**
Vermin, bird control	75.0 (18.7)	67.0 (20.7)	0.11
Location, environment	58.0 (27.6)	20.0 (22.1)	**< 0.01**
Internal biosecurity			
Management diseases	55.1 (22.0)	49.8 (19.1)	0.33
Farrowing, suckling period	69.2 (16.2)	75.3 (18.0)	0.17
Nursery period	78.5 (16.0)	77.9 (19.0)	0.91
Fattening period	40.4 (13.2)	42.5 (17.0)	0.59
Compartmentalizing, working lines, equipment	43.0 (20.2)	45.6 (18.2)	0.56
Cleaning, disinfection	74.2 (8.2)	66.2 (8.1)	**< 0.01**

^3^Applied level of significance 5% (*p* < 0.05). Significant differences are highlighted in black and bold

^a^*n* Number

^b^*SD* Standard deviation

**Table 5:**
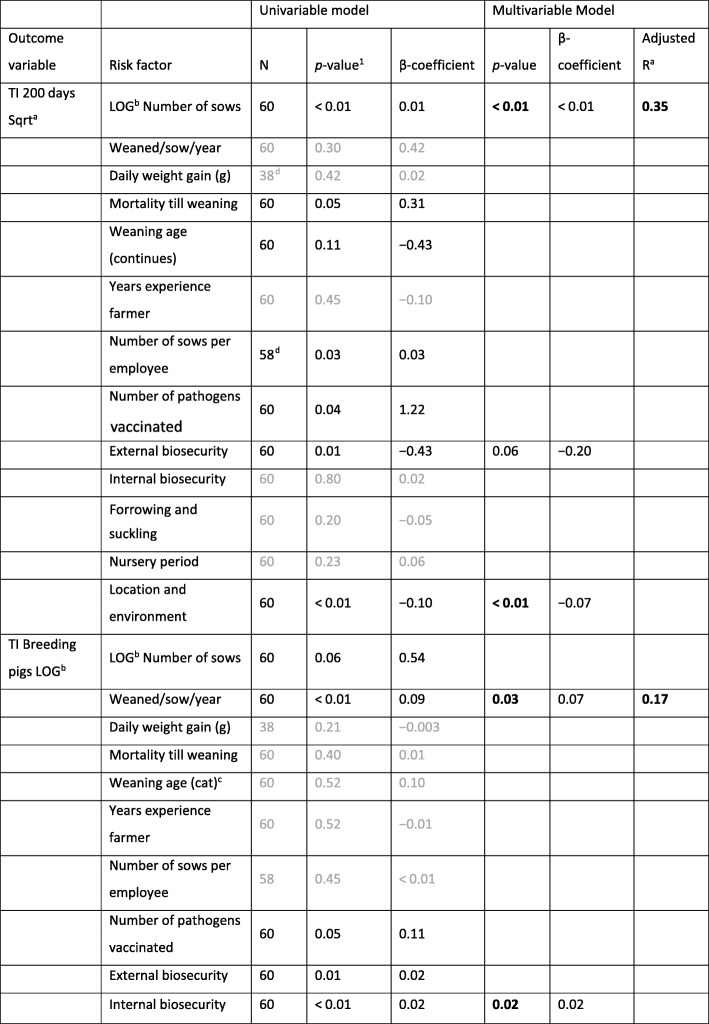
Statistical results of univariable and multivariable linear models for the outcomes TI 200 days and TI breeding pigs

^1^Light gray values in the univariable model indicate that these factors were not significant (*p* < 0.20) in the univariable model. In the multivariable model the *p*-values which are significant with *p* < 0.05 are black and bold, 0.05 < *p* < 0.10 are black and *p* > 0.10 are light gray

^a^*Sqrt* Square root transformation

^b^*LOG* Log transformation

^c^*Cat* Categorical variable

^d^Data was not available in all participating herds

## Discussion

### Study design

The objective of the study was to describe the AMU in pig herds and to identify herd management and biosecurity characteristics influencing this usage. To identify risk factors associated with a higher AMU in the pig sector, our field study focussed on farrow-to-finish farms. It has to be pointed out, that in observational studies of this type (cross-sectional) the levels of usage might be subject to selection bias. The participation depended on voluntary basis and maybe these farmers were in general more interested and hence representing the better performing herds. Moreover, we have to consider, that the obtained associations are the results of a cross-sectional study, not allowing to make direct causal conclusions. The data and information collected was reviewed and checked using visual inspection and documentation to minimize recall bias.

The average herd size (number of breeding pigs) in our sampled herds was 396 (median 300) sows per herd. Nationally the average number of breeding pigs (sow, gilts and boars) per farm is 145 [22]. This suggested that our sample population represented the larger herds. Most of the herds were located in Niedersachsen and Nordrhein-Westfalen where the average pig density is > 179 pigs/km^2^ and > 209 pigs/km^2^ respectively which is above the national average (75 pigs/km^2^) [24]. These criteria (volunteer farmers, herd size and pig density) resulted in a possible participation bias. On the other hand, the participating herds were located in regions where the majority of the German pig production takes place, suggesting that the results gave a good insight of the associations between pig production, biosecurity and AMU in German farrow-to-finish farms.

### Antimicrobial usage and disease incidence

To be able to compare the differences in the AMU in an objective manner a standardised approach was needed. To interpret the amount of consumed antimicrobials it is not sufficient to rely on data on purchased antimicrobials in kilogram [14, 17]. The provision of AMU data via pharmacies or other central dispensary locations is not suitable for Germany. Due to the legal compliance the application and dispensing records provided by the farmer or veterinarian yielded reliable data on the AMU on herd level [23]. Commercial in-feed medication was not recorded and applied in the participating farms. Only very few feed mills have the manufacturing authorisation to produce commercial in-feed medication [25]. The production is complex and cost-intensive and accordingly this method to apply antimicrobials in Germany is almost obsolete [25]. Several studies investigated different methods for the quantification of antimicrobial consumption [16, 26, 27]. In this study the treatment incidence based on a DDDA which is also proposed by the ESVAC consortium was applied [16, 21, 28]. The method allows the comparison between farms, regions and countries and is based on the dose and weight of the pharmacologically active ingredient. The DDDAs used in this study were developed to quantify the amount of antimicrobials administered to the pigs. The most transparent method to establish a consensus DDDA was to take the mean of all authorized products with the same active ingredient and administration route [21]. Most of the treatments were used in the youngest animals, the suckling and weaned pigs. Particularly in these age groups a large between-farm variance has been identified. These differences of the amount of antimicrobials used in suckling and weaned pigs were also described in other countries, including Belgium and France [16–18]. Furthermore, it was suggested, that this variance between the farms was related to differences of disease incidence, management factors, biosecurity measures and socioeconomic factors, like farmers’ and veterinarians’ attitude [29–31].

Regarding antimicrobial classes considered to be especially critical to human medicine, we identified a relatively frequent usage of third generation cephalosporines in suckling pigs. These products promise advantages to the farmer, because they are usually long and potent acting and can be administered in lower doses [15]. They were most likely applied to suckling pigs after routine manipulation such as castration and teeth clipping. Similar to our findings, in the study of Callens et al. [5] the use of more modern antimicrobials (e.g. cephalosporines) increased. Yet these products belong to the most critical important antimicrobials according to the WHO list. Therefore, it is highly unwanted to see such a high and increasing use. In some European countries (e.g. Belgium, The Netherlands, Denmark) the use of these third and fourth generation cephalosporines in pigs has been highly regulated or even banned [9, 32, 33]. The most common indication for a treatment in suckling and weaned pigs were disease symptoms related to the gastro-intestinal and locomotive system (e.g. lameness). The frequent use of aminopenicillins, macrolides and polymyxines in these age groups indicated, that these symptoms were most likely treated with these antimicrobial classes. Especially the frequent application of macrolides and polymyxines (mainly colistin) is rather worrying, since in the ‘Guidelines for the prudent use of antimicrobials in veterinary medicine’ it is recommended to reduce both macrolides and colistin [34]. Moreover, the recent discovery of plasmid-mediated resistance to colistin via the genes *mcr-1*, *mcr-2, mcr-3, mcr-4* and *mcr-5*, even more demand for a restrictive use [35–38]. Farmers reported, that fattening pigs were commonly treated against respiratory symptoms. The high amounts of aminopenicillins in this age group presumed a frequent use of this antimicrobial class. Diseases of the reproductive tract in breeding pigs were mainly treated with tetracyclines. Moreover, farmers described, that treatments against disorders of the locomotive system in breeding pigs increased since 2013. According to the ‘Animal Protection Keeping of Production Animals Order’ (TierSchNutztV) group housing of sows is obligatory since January 2013 [39]. Farmers observed increased ranking fights in the groups after weaning of the piglets. Hence, they could have reported more treatments and losses due to disorders of the locomotive system. When analysing the treatment records in our study, especially the antimicrobials applied to the suckling and weaned pigs were administered regularly assuming reoccurring clinical diseases in these age groups and thus resembling treatments in a preventive manner. Callens et al. [5] also reported, that the majority of strategic group treatments in pigs are given during the suckling and nursery period. The prophylactic use (treatment of healthy animals) of antimicrobials must be avoided in Germany [34]. Moreover, the median TI in the weaned pigs (TI = 487.6) indicates, that animals were treated 49% of the days in the nursery period. A TI over 1000, which was found in 19 out of 60 farms, implies that the animals were treated 1000/1000 days, or 100% of their lifespan/period duration. Accordingly, the treatment method resembles more a preventive rather than a metaphylactic (treatment of clinically healthy animals belonging to the same group or pen as animals with clinical symptoms [40]) or curative treatment.

### Biosecurity status

The prevention of the introduction of porcine pathogens into pig herds is still a challenging task for farmers and herd veterinarians. Biosecurity is an important tool to maintain the health status of pig herds [41]. The assessment of the level of biosecurity of the participating herds and the derived information was used to analyse the associations between parameters (biosecurity level, management parameters and AMU) measured over the preceding twelfth months. In general, it could not be ruled out, that certain biosecurity measures have changed over the preceding year before the herd visit, e.g. due to disease outbreaks, so that the scores might be different from the average at the point of the data collection. Compared to the overall score for external biosecurity (measures to prevent the introduction of pathogens into a herd from outside), the score for internal biosecurity (measures to reduce the spread of pathogens within the herd) was lower, which is in line with the findings of Laanen et al. [18]. Other studies showed that the level of biosecurity is influenced by the herd size, suggesting that larger herds tend to have a higher biosecurity status [18, 42–44]. According to Laanen et al. [18] it is easier for larger farms to implement a higher level of external and internal biosecurity which might help to achieve a lower level of AMU. In general, it is suggested, that an improved biosecurity leads to a better health status and thus to a reduced need of antimicrobials. But in contrast, the obtained score for internal biosecurity in our study was positively associated with the treatment incidence (TI breeding pigs). One possible explanation for this association could be the intensified improvement of internal biosecurity measures due to a current high infection pressure in the herds which led to a higher AMU. Furthermore, farmers with a higher internal biosecurity score might follow an overall precautionary principle and are prone to a faster antimicrobial treatment. It could also be suggested, that on larger farms more attention is paid to the internal biosecurity subcategories like disease management, working lines, compartmentalizing etc. These findings were previously reported by other studies [18, 42–44]. The negative association between the subcategory “location and environment” (external biosecurity) and the treatment incidence of pigs from birth till slaughter emphasized the importance of implementing biosecurity measures to prevent pathogens to enter a herd.

### Risk factors associated to a higher antimicrobial usage

The aim of this study was to describe and analyse risk factors for a high AMU in German farrow-to-finish farms. In order to explore farms with a higher usage in detail, we compared farms below (group 1) and above (group 2) the median TI 200 days. We observed, that farms belonging to group 2 most likely applied more antimicrobial treatments throughout the whole production line (from suckling to fattening pig) and not to one specific age group. Based on experience, most of the farmers know the critical time points, when their pigs get ill. At these critical time points farmers applied more antimicrobial treatments [5, 16]. But in contrast, these obvious differences were not observed in the treatment incidence in breeding pigs. The results showed, that breeding pigs received less treatments compared to growing pigs. Thus, the observed differences in the treatment incidence might be more pronounced in the suckling, weaned and fattening pigs. Significant differences between group 1 and 2 were observed in the biosecurity scores. A risk factor for a higher AMU was identified for farms, which were located in densely populated pig areas and tended to have worse biosecurity practices for visitors and personnel (external biosecurity). This is in accordance to the study by Collineau et al. [45], who described the profile of ‘top-farms’ in terms of low AMU and high technical performance. Direct association of the level of external biosecurity and AMU was described in Postma et al. [46], where a better external biosecurity was associated with a lower AMU in pigs from birth till slaughter. This finding was confirmed in our study where a nearly significant association between the level of external biosecurity and TI 200 days (*p* = 0.06) also suggest that a lower score for external biosecurity results in more antimicrobial treatments for pigs from birth till slaughter. The introduction of pathogens from an outside source increases the risk of disease onset in pig husbandry [44–46]. Since external biosecurity reduces the risk of entrance of pathogens into a herd and exit of pathogens out of a herd, a higher level of protection might reduce the disease pressure and thus reduce the need for antimicrobial treatments. Other studies demonstrated a strong correlation of external and internal biosecurity [47] and a reduced need for antimicrobial treatments in herds with a better internal biosecurity [18]. We observed, that farms above the median TI 200 days also scored lower in the subcategory ‘cleaning and disinfection’. The correct procedure of cleaning and disinfecting compartments and materials will reduce the risk of transmitting pathogens, which was already described for several pathogens like *Salmonella spp*., *Campylobacter spp*. and *Listeria monocytogenes* [48, 49]. In a study by Laanen et al. [18], a better score for internal biosecurity resulted in a lower AMU. Thus the improvement of internal biosecurity on herd level (e.g. strict hygiene protocol, all-in-all-out practice) is a rather simple option to reduce the necessity to use antimicrobials [50]. But in contrast we observed a significant negative association between internal biosecurity and AMU in breeding pigs. We have no explanation for this finding, but as mentioned above, the total AMU in breeding pigs was substantially lower than in growing pigs. Since the majority of antimicrobial treatments were used in the growing pigs, where we identified a nearly significant positive association between external biosecurity and AMU, more weight is given to this association. Thus, to aim at a reduced AMU, the use of antimicrobials in the growing phase would need to be addressed first. The multivariable linear regression model highlighted, that the TI 200 days was significantly higher in herds with more breeding sows present. Herds with more breeding sows usually work in a one-week-batch-farrowing-system to keep smaller groups and reduce the workload peaks in the more intensive periods around farrowing. In this production rhythm the piglets are usually weaned at a lower age (average of 21 days). The process of weaning introduces a number of stress factors that may influence the immune function and intestinal microflora of weaned pigs. These disturbances might challenge the risk of enteric disorders such as post-weaning colibacillosis (PWC), which is mainly caused by enterotoxigenic *Escherichia coli* (ETEC) [51]. Hence it could be suggested, that weaning at a lower age increases the risk for infection due to an immature enteric microflora. Weaning earlier than 21 days of age is banned in the EU and the legislation might enforce a minimum of 28 days [52]. According to Postma et al. [46] a higher weaning age was associated with a lower necessity for antimicrobial therapy. Moreover, one of the several factors determining the differences in AMU between countries might be related to the differences in the weaning age. Scandinavian countries like Sweden wean the pigs closer to 35 days of age and have a low usage of antimicrobials compared to other EU countries [1].

We showed that farms with a higher treatment incidence for breeding pigs significantly weaned more piglets per sow and year (WSY). One possible explanation could be the more active involvement of the farmer during the farrowing process. Since that is the period of antimicrobial treatments for sows normally, the survival rate of the piglets increased, resulting in more weaned pigs per sow and year [53, 54]. Another possible explanation is an increased sensitivity to disease as high productive sows might require more antimicrobial treatments as suggested by Postma et al. [46]. Moreover Postma et al. [46] suggested, that the farrowing rate might increase due to a positive effect of AMU on the incidence of mastitis and endometritis. A good health status of the sow might also improve the nursing abilities. Thus, the transfer of maternal derived antibodies to the piglets is optimized.

## Conclusion

In conclusion this study conducted the first attempt to identify associations between AMU and herd-level management factors in 60 German farrow-to-finish farms. Suckling and weaned pigs received the most antimicrobial treatments and were mainly treated with macrolides and aminopenicillines, respectively. Antimicrobial substances were commonly administered orally. Biosecurity scores showed large variations between the studied herds. Compared to the internal biosecurity score, higher scores were obtained for the external biosecurity. Farms with a high AMU in pigs from birth till slaughter generally accounted for a higher AMU in suckling, weaned and fattening pigs. Moreover, they were located in a less favorable region with a high pig density, had less strict hygiene regulations on visitors and personnel and a worse cleaning and disinfection regime compared to farms with a lower usage. Thus, the improvement of biosecurity practices could be an important tool to reduce the necessity of AMU. For herds with a higher treatment incidence in breeding pigs and pigs from birth till slaughter we identified associated risk factors like the herd size and biosecurity status. Farms with a higher AMU might be more strictly adhering to routine habits to treat their animals. Breaking the routines trough guidance of a prudent AMU might reduce the necessity of antimicrobial treatments. The results of this study suggest that the main focus for reducing AMU should be on growing pigs and farms with a high AMU since this most likely will have the best effect. This enhanced knowledge of risk factors for a higher AMU might support and specify farm advice and policy making towards a more prudent use of antimicrobials.

## Additional file

Additional file 1: Descriptive information on the frequency of treatments against predefined disease symptoms per age category. (DOCX 16 kb)

## Abbreviations

AMU: Antimicrobial Usage
ATC: Anatomical Therapeutic Chemical
DDD: Daily defined dose
DDDA: Defined daily dose animal
e.g.: Exempli gratia (for example)
EFSA: European Food Safety Authority
EMA: European Medicines Agency
ESVAC: European Surveillance Veterinary Antimicrobial Consumption
etc: Et cetera
ETEC: Enterotoxigenic *Escherichia coli*
EU: European Union
g: Gramm
i.e.: Id est
kg: Kilogram
km: Kilometre
l: Liter
LOG: Logarithmic
mg: Milligram
ml: Millilitre
OIE: World Organisation for Animal Health
PRRSV: Porcine Respiratory and Reproductive Syndrome Virus
PWC: Post-weaning colibacillosis
SD: Standard deviation
Sqrt: Square root
t: Tonnes
TI: Treatment incidence
WHO: World Health Organisation
WSY: Weaned piglets per sow and year
