# Supplementary material for: Association between antimicrobial usage, biosecurity measures as well as farm performance in German farrow-to-finish farms
Source: Porcine Health Manag. 2018 Dec 14;4:30. doi: 10.1186/s40813-018-0106-5 (PMC6293545; doi:10.1186/s40813-018-0106-5)
Supplement: Supplementary file 1 — Descriptive information on the frequency of treatments against predefined disease symptoms per age category. (DOCX 16 kb) [file 40813_2018_106_MOESM1_ESM.docx]

**Additional file 1** **Descriptive information on the frequency of treatments against predefined disease symptoms per age category**

| Scale | | | | | |
| --- | --- | --- | --- | --- | --- |
|  | 1  (never) | 2  (rarely) | 3  (occasionally) | 4  (regularly) | 5  (always) |
| Count  (Proportion) | | | | | |
| **Suckling pigs** |  | | | | |
| lameness | 4 (7%) | 22 (37 %) | 25 (42%) | 8 (14%) | 0 (0%) |
| gastro-intestinal | 4 (7%) | 26 (43%) | 17 (28%) | 12 (20%) | 1 (2%) |
| respiratory | 29 (48%) | 27 (45%) | 2 (3%) | 2 (3%) | 0 (0%) |
| nervous | 17 (28%) | 31 (52%) | 10 (17%) | 2 (3%) | 0 (0%) |
| skin | 24 (40%) | 29 (48%) | 6 (10%) | 1 (2%) | 0 (0%) |
| **Weaned pigs** |  | | | | |
| lameness | 2 (3%) | 23 (38%) | 30 (50%) | 4 (7%) | 1 (2%) |
| gastro-intestinal | 6 (10%) | 17 (28%) | 17 (28%) | 16 (27%) | 4 (7%) |
| respiratory | 4 (7%) | 20 (33%) | 29 (48%) | 6 (10%) | 1 (2%) |
| nervous | 0 (0%) | 24 (40%) | 31 (52%) | 5 (8%) | 0 (0%) |
| skin | 3 (5%) | 26 (43%) | 22 (37%) | 8 (13%) | 1 (2%) |
| **Fattening pigs** |  | | | | |
| lameness | 3 (5%) | 39 (65%) | 16 (27%) | 2 (3%) | 0 (0%) |
| gastro-intestinal | 35 (59%) | 20 (34%) | 3 (5%) | 1 (2%) | 0 (0%) |
| respiratory | 9 (15%) | 30 (50%) | 17 (28%) | 3 (5%) | 1 (2%) |
| nervous | 29 (49%) | 27 (46%) | 3 (5%) | 0 (0%) | 0 (0%) |
| skin | 7 (12%) | 32 (53%) | 17 (28%) | 3 (5%) | 1 (2%) |
| **Breeding pigs** |  | | | | |
| lameness | 1 (2%) | 28 (47%) | 27 (45%) | 4 (7%) | 0 (0%) |
| gastro-intestinal | 49 (82%) | 11 (18%) | 0 (0%) | 0 (0%) | 0 (0%) |
| respiratory | 15 (25%) | 38 (63%) | 6 (10%) | 1 (2%) | 0 (0%) |
| nervous | 49 (82%) | 11 (18%) | 0 (0%) | 0 (0%) | 0 (0%) |
| skin | 17 (28%) | 35 (58%) | 8 (13%) | 0 (0%) | 0 (0%) |
| reproductive | 6 (10%) | 32 (53%) | 18 (30%) | 3 (5%) | 1 (2%) |
| mastitis | 1 (2%) | 29 (48%) | 23 (38%) | 7 (12%) | 0 (0%) |
